# Design of a Phase 3, Multicenter, Randomized, Open-Label Study of Nipocalimab or IVIG and Prednisone in Pregnancies at Risk for Fetal and Neonatal Alloimmune Thrombocytopenia

**DOI:** 10.1055/a-2753-9323

**Published:** 2025-12-12

**Authors:** James Bussel, Barbara Stegmann, Pamela Baker, Abbie Oey, Yanxin Jiang, Rebecca Zaha, Hillary Van Valkenburgh, Babajide Keshinro

**Affiliations:** 1 Department of Pediatrics, Division of Hematology/Oncology, Weill Cornell Medical College, New York, New York, United States; 2 New York Presbyterian Hospital, New York, New York, United States; 3 Johnson & Johnson, Spring House, Pennsylvania, United States; 4 Johnson & Johnson Biologics BV, Leiden, the Netherlands

**Keywords:** neonatal Fc receptor blocker, FcRn, FNAIT, safety, efficacy, study design, thrombocytopenia

## Abstract

**Objective:**

Nipocalimab, a neonatal Fc receptor blocker, showed evidence of efficacy and safety in preventing or delaying fetal anemia in a phase 2 study of early-onset severe hemolytic disease of the fetus and newborn, demonstrating potential for treatment of other maternal immunoglobulin G alloantibody-mediated fetal diseases. The phase 3 FREESIA-3 study aims to evaluate the efficacy and safety of nipocalimab or intravenous immunoglobulin (IVIG) with prednisone in pregnancies with a previous occurrence of fetal and neonatal alloimmune thrombocytopenia (FNAIT) with or without intracranial hemorrhage or severe fetal/neonatal bleeding (high- or standard-risk, respectively).

**Study Design:**

FREESIA-3 is a phase 3, open-label, randomized, multicenter study in pregnant individuals at risk for FNAIT. Participants are randomized 4:1 to receive either weekly 45 mg/kg intravenous nipocalimab or weekly IVIG with prednisone starting at 13 to 18 weeks of gestational age (standard-risk) or 12 weeks of gestational age (high-risk) until delivery. During treatment, pregnant participants will receive ultrasound monitoring every 2 weeks for fetal bleeding, growth, and development. Postnatal follow-up is 24 weeks for maternal participants and 104 weeks for neonates/infants.

**Results:**

The primary endpoint is an adverse outcome of death or adjudicated severe bleeding in utero up to 1 week postbirth, or platelet count at birth of < 30 × 10 ^9^ /L in a fetus/neonate. Secondary endpoints include fetal/neonatal death, neonatal platelet count at birth, nadir neonatal platelet count over 1 week postbirth, neonate requiring platelet transfusion(s), adjudicated fetal and neonatal bleeding up to 1 week postbirth, neonate receiving IVIG for thrombocytopenia, safety in maternal participants and neonates/infants, and immunogenicity of nipocalimab. Exploratory endpoints include patient- and caregiver-reported outcome assessments and nipocalimab pharmacokinetics and pharmacodynamics.

**Conclusion:**

FREESIA-3, an open-label, multicenter, randomized, phase 3 study, will evaluate the efficacy and safety of nipocalimab in both standard- and high-risk pregnancies for FNAIT.

**Key Points:**

- FNAIT is a transplacental alloantibody-driven disease.
- Nipocalimab blocks IgG recycling, lowering IgG levels.
- Nipocalimab blocks IgG placental transfer to the fetus.
- Nipocalimab reduced adverse outcomes in EOS-HDFN.
- FREESIA-3 studies nipocalimab efficacy/safety in FNAIT.

## Introduction

Fetal and neonatal alloimmune thrombocytopenia (FNAIT) is a rare but serious pregnancy complication in which maternal exposure to incompatible, paternally derived human platelet antigens (HPAs) leads to immunization and generation of alloantibodies. During FNAIT-affected pregnancies, maternal immunoglobulin G (IgG) alloantibodies directed against HPAs cross the placenta via neonatal Fc receptor (FcRn)-mediated transport, binding to fetal platelets and megakaryocytes and leading to platelet destruction and impaired production, and thrombocytopenia. 1 2 3 The transfer of maternal IgG to the fetus is minimal during the first trimester, but rises progressively in the second and third trimesters. 4 HPA-1a is the most common HPA subtype in Caucasians and is responsible for 80 to 85% of FNAIT cases, followed by HPA-5b, which accounts for 7 to 16% of FNAIT cases. 1 5 6 7 Other HPA subtypes, such as HPA-9b, HPA-3a, and HPA-3b, may be underreported due to difficulties with alloantibody testing. 6 8 9

FNAIT occurs with an incidence of approximately 1 per 1,000 to 2,000 pregnancies. 1 10 While many FNAIT cases are mild, it can be life-threatening when intracranial hemorrhage (ICH) develops in the fetus or neonate, which often leads to death or severe neurologic damage. 2 3 10 11 In pregnancies with a previous occurrence of FNAIT without ICH or severe fetal/neonatal bleeding (defined as standard-risk FNAIT), the likelihood of ICH is significantly lower compared with affected pregnancies that had a prior history of FNAIT accompanied by ICH (defined as high-risk FNAIT). 12 Recent studies have shown that even in cases of FNAIT without an ICH, children of affected pregnancies may suffer long-term neurologic impairments. 13 14 These events are hypothesized to be caused by placental inflammation and are potentially preventable by effective FNAIT treatment. Following the birth of an infant affected by FNAIT, the likelihood of recurrence in subsequent fetal antigen-positive pregnancies is substantially elevated, depending upon paternal genetics for the HPA-1a/b locus. 15

Administration of intravenous immunoglobulin (IVIG), with or without prednisone, is the standard of care for antenatal treatment of FNAIT. 2 11 However, IVIG poses a demand on the health care system and patients, including resource utilization, tolerability issues (e.g., headaches, flu-like symptoms), and patient burden (e.g., long infusion times). 16 17 18 There are no universally recommended dosing regimens for IVIG. 19 In the United States, 1 to 2 g/kg IVIG with prednisone is used, 20 whereas in many other countries, 0.5 to 1 g/kg IVIG without prednisone is common, 7 19 21 22 and in some European countries (e.g., Norway), IVIG use is limited to high-risk (previous ICH) pregnancies and those at standard-risk with high anti-HPA-1a antibody levels. 2 18 21 Recently, an international consensus on FNAIT management developed by the FNAIT Expert Group, using a modified Delphi analysis, concluded that corticosteroids should not be routinely administered in standard-risk HPA-1 alloimmunized pregnancies, although this has not been universally adopted, and there was no consensus on whether corticosteroids should be used in conjunction with IVIG in high-risk pregnancies. In addition, a consensus was not reached on the need for treatment of standard-risk HPA-5b-alloimmunized pregnant individuals in subsequent pregnancies. 12 While weekly administration of 1 g/kg IVIG increases the fetal platelet count in more than 50% of cases, two randomized studies demonstrated that, in severe fetal thrombocytopenia (e.g., < 20,000/µL), IVIG 1 g/kg alone was not effective in substantially or rapidly increasing platelet count. Adding prednisone to the IVIG treatment regimen led to a statistically significant increase in platelet count. 23 24 IVIG is thought to create its effect primarily by saturating FcRn, thus lowering maternal anti-HPA-1a levels and preventing its transfer to the fetus. 25

**Fig. 1:**
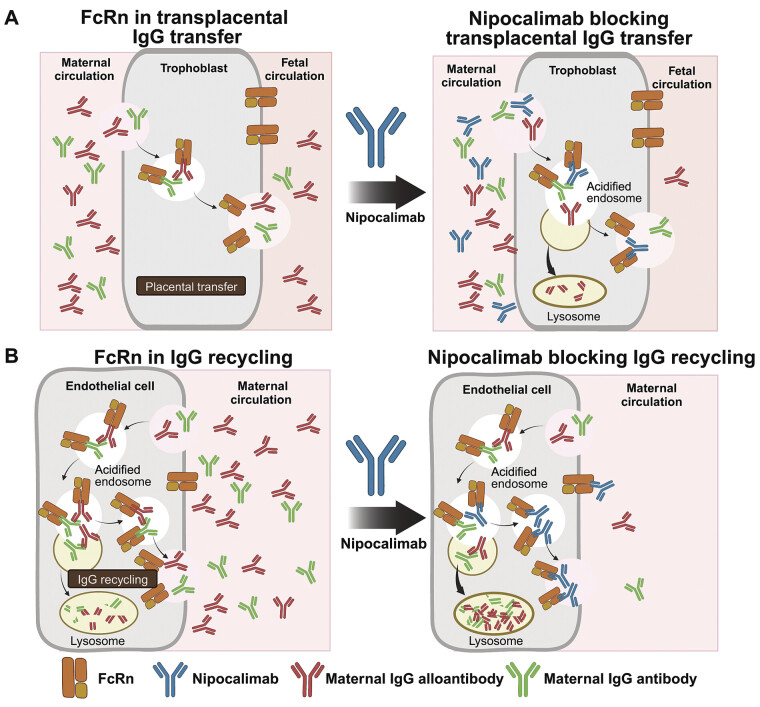
Anticipated prevention of FNAIT by nipocalimab through ( **A** ) blocking transplacental transfer and ( **B** ) IgG recycling in maternal circulation. FcRn, neonatal Fc receptor; FNAIT, fetal and neonatal alloimmune thrombocytopenia; HPA, human platelet antigen; ICH, intracranial hemorrhage; IgG, immunoglobulin G. Reproduced with permission from Massachusetts Medical Society. 30

As the functions of FcRn are to transfer maternal IgG across the placenta and to maintain the long half-life of serum IgG, blocking FcRn may improve outcomes in diseases caused by pathogenic IgG. 26 27 28 29 Nipocalimab is a fully human, IgG1, high-affinity, aglycosylated, FcRn-blocking monoclonal antibody that inhibits transplacental IgG transfer and lowers circulating maternal IgG levels ( Fig. 1 ). 30 31 Nipocalimab showed proof-of-concept efficacy in preventing or delaying fetal anemia with an acceptable safety profile in an open-label phase 2 study of early-onset severe hemolytic disease of the fetus and newborn (EOS-HDFN; ClinicalTrials.gov Identifier: NCT03842189). In this study, 54% (7/13) of pregnant participants treated with nipocalimab had live births at ≥ 32 weeks gestational age (GA) without needing an intrauterine transfusion (IUT), significantly surpassing the historical benchmark of 10% ( *p* < 0.001). Additionally, 46% (6/13) of pregnancies treated with nipocalimab did not require either IUTs or postnatal transfusions. Consistent with its mechanism of action, nipocalimab treatment led to decreases in alloantibody titer and IgG levels in maternal and cord blood samples, with no reports of unusual maternal or pediatric infections. 30 These findings in EOS-HDFN support the potential of nipocalimab for the treatment of other IgG alloantibody-mediated perinatal diseases, including the ongoing phase 3 clinical development program of nipocalimab in FNAIT (FREESIA-1 32 : ClinicalTrials.gov Identifier: NCT06449651 and FREESIA-3: ClinicalTrials.gov Identifier: NCT06533098).

Herein, we outline the design of the open-label FREESIA-3 study that aims to assess the efficacy and safety of nipocalimab with a contemporaneous reference arm of IVIG with prednisone in pregnancies at standard-risk or high-risk for FNAIT.

## Materials and Methods

### Ethics

The FREESIA-3 trial follows International Council for Harmonisation guidelines on Good Clinical Practice 33 and applicable regulatory and local requirements. Each participating center received approval from its independent ethics committee/institutional review board according to applicable national regulations. All participants provide their consent to participate in the study after receiving details of study procedures, risks, and requirements of the study, and are updated on any new information that could influence their decision to continue. Participation is voluntary and may be withdrawn at any time without penalty or loss of benefits.

### Participants

Eligible participants are 18 to 45 years of age, are pregnant with an estimated GA of 13 to 18 weeks, and their current pregnancy includes maternal anti-HPA-1a and/or anti-HPA-5b alloantibody and an HPA-1a- and/or HPA-5b-positive fetus. To be eligible, pregnant individuals must have a history of FNAIT in ≥ 1 prior pregnancy with (1) neonatal platelet count < 150 × 10 ^9^ /L with no fetal/neonatal ICH or other severe fetal/neonatal hemorrhage (standard-risk of FNAIT) or (2) fetal/neonatal ICH or severe bleeding in a previous child (high-risk of FNAIT; see complete inclusion criteria in Table 1 ). The inclusion of participants with high-risk FNAIT pregnancies in FREESIA-3 will be based on the recommendation from the Data Monitoring Committee after reviewing an interim analysis of the placebo-controlled FREESIA-1 study, which is evaluating the safety and efficacy of nipocalimab in standard-risk FNAIT pregnancies. 32

**Table 1:** Completed inclusion and exclusion criteria

**Inclusion criteria**
• Female aged 18–45 y at the time of informed consent • Pregnant and an estimated GA of 13 ^0/7^ to 18 ^6/7^ wk • History of ≥ 1 prior pregnancy with FNAIT (based on medical record review), including: ○ Neonatal platelet count < 150 ×10 ^9^ /L with no fetal/neonatal ICH or severe fetal/neonatal hemorrhage (standard-risk), or ○ Fetus/neonate with ICH or severe hemorrhage in a previous child based on medical records (high-risk) • Current pregnancy with the presence of maternal anti-HPA-1a and/or anti-HPA-5b alloantibody and positive fetal HPA-1a and/or HPA-5b genotype • Health status considered stable by the investigator on the basis of physical examination, medical history, vital signs, 12-lead ECG, and clinical laboratory tests performed at screening • Must sign an informed consent form indicating that the maternal participant understands the purpose of and the procedures required for the study and is willing to participate in the study for up to 24 wk of follow-up. The parent(s)/guardian(s) (preferably both if available, or as per local requirements) of the neonate/infant must sign an informed consent form indicating that they understand the purpose of and the procedures required for the study and agree to permit 104-wk follow-up for the neonate/infant and to complete caregiver-reported outcomes for the infant • Willing to forego participation in another clinical study of an investigational therapy for the duration of participation of the maternal participant and her neonate/infant in the current study • Must agree not to donate blood through the final follow-up visit at week 24 postpartum
Exclusion criteria
• Currently pregnant with multiple gestations (twins or more) • History of severe preeclampsia in a previous pregnancy • History of severe fetal growth restriction (birth weight < third percentile for GA) in a previous pregnancy • History of myocardial infarction, unstable ischemic heart disease, or stroke • History of thrombosis or risk factors of developing thrombosis at any time, or history of unprovoked pulmonary embolism • History of renal insufficiency or has a risk of developing renal dysfunction or acute renal failure • Known allergies, hypersensitivity, or intolerance to nipocalimab or its excipients, to IVIG, or to prednisone • History or current diagnosis of autoimmune thrombocytopenia • History of fetuses or neonates with major congenital abnormalities or chromosomal abnormalities • History of IgA deficiency with anti-IgA antibodies • Has a condition of hyperprolinemia • History of severe, progressive, and/or uncontrolled hepatic (e.g., viral/alcoholic/autoimmune hepatitis and/or metabolic liver disease), gastrointestinal, renal, pulmonary, cardiovascular, psychiatric, neurological, or musculoskeletal disorder; hypertension; and/or any other medical or uncontrolled autoimmune disorder(s) (e.g., diabetes mellitus) or clinically significant abnormalities in screening laboratory values that might interfere with the participant's full participation in the study or confound the protocol-specified assessments, and/or that might jeopardize the safety of the participant or her fetus or the validity of the study results • Has any confirmed or suspected clinical immunodeficiency syndrome or has a family history of congenital or hereditary immunodeficiency, unless confirmed absent in the participant • History of solid organ or bone marrow transplantation (except for a corneal transplant performed ≥ 12 wk before screening) • Currently has a malignancy or has a history of malignancy within 3 y before screening (except for localized basal cell carcinoma and/or squamous cell skin cancer that has been adequately treated with no evidence of recurrence for ≥ 12 wk before the first study intervention administration or cervical carcinoma in situ that has been treated with no evidence of recurrence for ≥ 12 wk before the first study intervention administration) • Has shown a previous severe immediate hypersensitivity reaction, such as anaphylaxis to therapeutic proteins (e.g., monoclonal antibodies) • History of serious infection that required hospitalization or parenteral antibiotics within 8 wk of screening or a history of recurring severe infections • Has a severe infection, including opportunistic infections (e.g., pneumonia, biliary tract infection, diverticulitis, Clostridium difficile infection, cytomegalovirus, pneumocystosis, aspergillosis) requiring parenteral anti-infectives or hospitalization, or is assessed as serious/clinically significant by the investigator, within 8 wk prior to screening • Has a severe chronic infection (e.g., bronchiectasis, chronic osteomyelitis, chronic pyelonephritis) or requires chronic treatment with anti-infectives (e.g., antibiotics, antivirals) • COVID-19 infection: has tested positive for or been exposed to COVID-19 within 4 wk prior to the first dose of study intervention. Participants who have tested positive for or been exposed to COVID-19 may participate if they have both an absence of symptoms and a negative validated COVID-19 test obtained ≥ 2 wk after symptoms onset (or the first positive test for asymptomatic infection) or exposure. Follow local regulations/guidelines for validated COVID-19 testing procedures and the standard definition of COVID-19 exposure • Has received rituximab, eculizumab, or FcRn antagonists (e.g., efgartigimod) within 26 wk prior to screening • Is currently receiving systemic corticosteroids or other immunosuppressants ○ Use of low-potency topical corticosteroids, nasal/inhaled corticosteroids, or intra-articular corticosteroids is permitted • Has received or is currently receiving plasmapheresis, immunoadsorption therapy, or any IgG Fc-related therapeutics during the current pregnancy • Has received a live virus vaccination during the current pregnancy or has a known need to receive a live virus vaccination during the study while receiving study intervention or within ≥ 8 wk after the last administration of study intervention in this study • Has received a BCG vaccination within 1 y prior to the first dose of study intervention or has a known need to receive a BCG vaccine during the study or within ≥ 8 wk after the last administration of study intervention • Has previously received nipocalimab or was enrolled and received study intervention in this study in a previous pregnancy • Is currently enrolled or plans to enroll in an investigational study and receive investigational intervention during the study • Has received an investigational intervention within the period ≤ 5 half-lives of the investigational compound prior to screening or used an invasive investigational medical device within 12 wk prior to screening • History of any positive test for HIV at screening • Has positive laboratory test results for HBV infection • Has antibodies to HCV, unless they satisfy 1 of the following conditions: ○ Has a history of successful treatment, defined as being negative for HCV RNA ≥ 24 wk after completing antiviral treatment, and has a negative HCV RNA test result at screening ○ Has a negative HCV RNA test result > 24 wk prior to screening and a negative HCV RNA test result at screening • Has an active infection at screening or baseline with coxsackie, syphilis, cytomegalovirus, toxoplasmosis, parvovirus, or herpes simplex 1 or 2, as evidenced by clinical signs and symptoms or screening serology results from the central laboratory ○ Serology evidence of prior infection or exposure, but without clinical signs and symptoms of active infection, is acceptable to participate ○ Following discussion with the Sponsor, serology testing may be performed by an alternative certified laboratory while awaiting central laboratory serology results, if turnaround times delay receipt of test results prior to randomization • Has a screening laboratory test result of total IgG < 6 g/L • Has a screening laboratory test result of albumin < LLN • Has a screening laboratory test result of hemoglobin < 80 g/L • Has a screening laboratory test result of white blood cell count < 3.0 GI/L • Has a screening laboratory test result of absolute neutrophil count < 1.5 GI/L • Has a screening laboratory test result of platelet count < 100 GI/L • Has a screening laboratory test result of aspartate aminotransferase ≥ 2 × ULN ○ Values for ULN should be based on the normal reference range for the second trimester in pregnancy 50 • Has a screening laboratory test result of alanine aminotransferase ≥ 2 × ULN ○ Values for ULN should be based on the normal reference range for the second trimester in pregnancy 50 • Has a screening laboratory test result of estimated glomerular filtration rate < 90 mL/min per 1.73 m ^2^ • Has any condition in the current pregnancy (including known genetic defects of the fetus or umbilical cord abnormality) for which, in the opinion of the investigator, participation would not be in the best interest of the participant or fetus/neonate/infant (e.g., would compromise the well-being) or that could prevent, limit, or confound the protocol-specified assessments • History of moderate or severe substance or alcohol use disorder according to *Diagnostic and Statistical Manual of Mental Disorders, Fifth Edition* criteria, except nicotine and caffeine, within 1 y prior to screening and during the current pregnancy

Abbreviations: BCG, Bacillus Calmette-Guérin; ECG, electrocardiogram; FcRn, neonatal Fc receptor; FNAIT, fetal and neonatal alloimmune thrombocytopenia; GA, gestational age; HBV, hepatitis B virus; HCV, hepatitis C virus; HPA, human platelet antigen; ICH, intracranial hemorrhage; IgA, immunoglobulin A; IgG, immunoglobulin G; IVIG, intravenous immunoglobulin; LLN, lower limit of normal; ULN, upper limit of normal.

Exclusion criteria include multiple gestations in the current pregnancy; preeclampsia with severe features or severe fetal growth restriction (birth weight < third percentile for GA) in a previous pregnancy; history of myocardial infarction, unstable ischemic heart disease, stroke, thrombosis or risk factors of developing thrombosis, unprovoked pulmonary embolism, renal insufficiency, or risk of developing renal dysfunction or acute renal failure; history of serious infection requiring hospitalization or chronic treatment with anti-infectives; having serum total IgG < 6 g/L; previously receiving rituximab, eculizumab, or FcRn antagonists; currently receiving systemic corticosteroids or other immunosuppressants; history of or currently receiving plasmapheresis, immunoadsorption therapy, or any IgG Fc-related therapeutics; known allergies, hypersensitivity, or intolerance to nipocalimab or its excipients, IVIG, or prednisone (see complete exclusion criteria in Table 1 ).

### Study Design

**Fig. 2:**
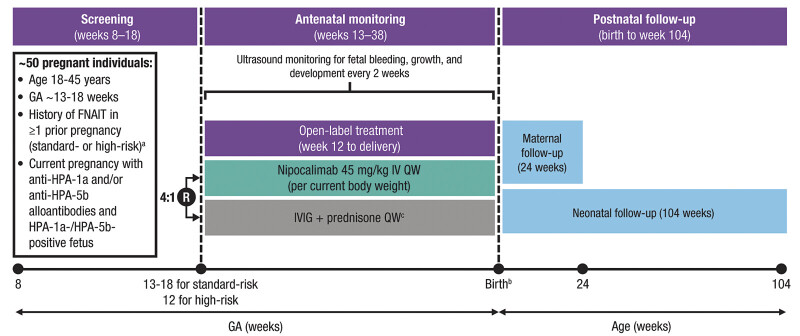
FREESIA-3 study design. FNAIT, fetal and neonatal alloimmune thrombocytopenia; GA, gestational age; HPA, human platelet antigen; IV, intravenous; IVIG, intravenous immunoglobulin; QW, weekly; R, randomization. ^a^ Standard-risk: neonatal platelet count < 150 × 10 ^9^ /L with no fetal/neonatal ICH or severe hemorrhage. High-risk: fetus/neonate with a history of ICH or severe hemorrhage in a previous child. ^b^ Delivery is planned to occur at 38 weeks, but may occur earlier at the discretion of the investigator and the study participant. ^c^ IVIG + prednisone dose regimen can be found in Table 2 .

FREESIA-3 is a phase 3, open-label, randomized, multicenter study in HPA-1a- and/or HPA-5b-alloimmunized pregnant individuals at standard-risk (no history of ICH in a previous pregnancy) or high-risk (history of ICH in a previous pregnancy) for FNAIT. Recruitment of 50 pregnant participants is planned from maternal-fetal medicine centers in Austria, Germany, Poland, the Netherlands, the United Kingdom, and the United States, starting with standard-risk FNAIT pregnancies. The study includes a screening period (GA weeks 8–18 for standard-risk and GA weeks 8–12 for high-risk participants), randomization (GA weeks 13–18 for standard-risk and GA week 12 for high-risk participants), open-label weekly treatment period of up to 29 weeks, and postnatal follow-up period (24 weeks for maternal participants and 104 weeks for infants; Fig. 2 ).

Maternal participants will be grouped into two cohorts by HPA incompatibility subtypes: the HPA-1a cohort or the HPA-5b cohort. Participants from each cohort will be separately randomized 4:1 to weekly nipocalimab (45 mg/kg IV) or weekly IVIG (1–2 g/kg) with daily prednisone (0.5–1 mg/kg; details of dose regimens are provided in Table 2 ) until delivery. Randomization will be balanced by using randomly permuted blocks for each cohort. For the HPA-1a cohort, participants will be stratified by FNAIT risk (standard- or high-risk). For the HPA-5b cohort, participants will not be stratified by risk due to the anticipated low number of participants with this subtype. IVIG and prednisone dosing follows the risk-stratified regimen adapted from Bussel et al. 34 The reference arm will provide contemporaneous information on outcomes associated with the current standard of care in the United States for pregnancies at risk for FNAIT. 20 During the treatment period, pregnant participants will receive ultrasound monitoring for fetal bleeding along with fetal growth and development assessments every 2 weeks. At birth and prior to hospital discharge, neonates will undergo cranial ultrasound scans for perinatal/neonatal ICH, have platelet count(s) assessed, and, as per protocol, if necessary, receive platelet transfusion and/or IVIG as appropriate.

**Table 2:** Dose regimen of IVIG and prednisone

Risk for FNAIT	Weeks of GA	Dose regimen of IVIG and prednisone	
Standard risk	20–31	• IVIG 1 g/kg QW and prednisone 0.5 mg/kg/d, or • IVIG 2 g/kg QW	
	32 to delivery	• IVIG 2 g/kg QW and prednisone 0.5 mg/kg/d	
	After delivery	• Prednisone tapering	
High risk		Previous fetus/neonate with ICH diagnosed at 28 wk of GA or later	Previous fetus/neonate with ICH diagnosed before 28 wk of GA
	12–19	• IVIG 1 g/kg QW	• IVIG 2 g/kg QW
	20–27	• IVIG 1 g/kg QW and prednisone 0.5 mg/kg/d, or • IVIG 2 g/kg QW	• IVIG 2 g/kg QW and prednisone 1 mg/kg/d
	28 to delivery	• IVIG 2 g/kg QW and prednisone 0.5 mg/kg/d	
	After delivery	• Prednisone tapering	

Abbreviations: FNAIT, fetal and neonatal alloimmune thrombocytopenia; GA, gestational age; ICH, intracranial hemorrhage; IVIG, intravenous immunoglobulin; QW, weekly.

### Study Assessments

A listing of study endpoints is provided in Table 3 . The primary composite endpoint is an adverse outcome of death or adjudicated severe bleeding (including ICH) in utero and up to the first week postbirth, or a platelet count at birth < 30 × 10 ^9^ /L in a fetus/neonate. An independent Bleeding Adjudication Committee will adjudicate all bleeding events. Secondary endpoints include adverse outcome of death of a fetus/neonate; platelet count at birth < 10, < 30, < 50, and < 150 × 10 ^9^ /L in a neonate; nadir neonatal platelet count in the first week postbirth; adjudicated fetal and neonatal bleeding up to the first week postbirth; neonate requiring platelet transfusion(s), including the number of platelet transfusions and the number of donor exposures for platelet transfusions; and neonate receiving IVIG for the treatment of thrombocytopenia.

**Table 3:** Study objectives and endpoints

Objectives	Endpoints
Primary	
• To assess the efficacy of the study intervention on the risk of severe FNAIT	• Adverse outcome of death or adjudicated severe bleeding in utero, and up to the first week postbirth, or platelet count at birth < 30 × 10 ^9^ /L in a fetus/neonate
Secondary	
• To assess the efficacy of the study intervention on FNAIT-related thrombocytopenia at birth	• Platelet count at birth in a neonate
• To assess the efficacy of the study intervention on the risk of FNAIT-related outcomes	• Adverse outcome of the death of a fetus/neonate • Platelet count at birth < 10, < 30, < 50, and < 150 × 10 ^9^ /L in a neonate • Nadir platelet count in a neonate over the first week postbirth • Neonate requiring platelet transfusion(s), including the number of platelet transfusions and the number of donor exposures for platelet transfusions • An adjudicated bleeding in utero, and up to the first week postbirth, in a fetus/neonate • A neonate requiring postnatal IVIG for the treatment of thrombocytopenia
• To evaluate the safety of the study intervention in maternal and neonatal/infant participants	• Maternal participant with a TEAE, SAE, or AESI • Maternal participant with a TEAE leading to discontinuation of study intervention • A neonate/infant with a TEAE, SAE, or AESI • A fetus/neonate with a TEAE of bleeding • A neonate with a TEAE of infection • Infant development as measured by Bayley Scales at weeks 52 and 104
• To assess the immunogenicity of nipocalimab in maternal participants who receive nipocalimab treatment	• Incidence of antibodies to nipocalimab, including neutralizing antibodies in maternal serum during pregnancy and postpartum
Exploratory	
• To assess the impact of the study intervention on pregnancy and neonatal outcomes	• Pregnancy resulting in preterm birth at < GA week 37 • Deliveries performed via c-section • Pregnancies affected by fetal growth restriction • Neonate with birth weight < 10th percentile of GA
• To assess the safety of the study intervention in maternal and neonatal/infant participants	• Change from baseline in laboratory parameters over time in maternal and neonatal/infant participants (e.g., infant's IgG)
• To assess the PK of nipocalimab in maternal participants who receive nipocalimab treatment	• Serum nipocalimab concentration in maternal blood over time during pregnancy and postpartum • Nipocalimab concentration in colostrum/breast milk (birth to 4 wk)
• To assess the PK of nipocalimab in the neonate born to maternal participants who receive nipocalimab treatment	• Nipocalimab concentration in the neonate over time
• To assess the PD of the study intervention in maternal participants and neonates/infants	• Total IgG in maternal and neonate/infant blood over time
• To assess the impact of the study intervention on health-related quality of life and health status in maternal participants and infants	• Maternal health-related quality of life: change from baseline in SF-36 v2 acute domains and component scores over time • Maternal health-related quality of life: change from baseline in EQ-5D-5L visual analog scale and EQ-5D-5L index scores over time • Infant health-related quality of life: IQI score over time
• To assess MRU data in neonates following antenatal study intervention	• Use of intensive care for neonates as measured by length of stay
• To assess the effect of the study intervention on the exploratory biomarkers in the maternal participants and neonates/infants	• Change from baseline in biomarker levels over time (e.g., alloantibodies as exploratory PD)
• To assess the effect of the study intervention on the placental findings	• Histopathologic placenta evaluation • Placenta weight and placenta/birth weight ratio (as a measure of placenta function)
• To evaluate the pharmacogenomics of the study intervention in maternal participants	• Genetic factors associated with the disease and clinical response

Abbreviations: AESI, adverse event of special interest; EQ-5D-5L, EuroQol 5-Dimension 5-Level; FNAIT, fetal and neonatal alloimmune thrombocytopenia; GA, gestational age; IgG, immunoglobulin G; IQI, Infant health-related Quality of life Instrument; IVIG, intravenous immunoglobulin; MRU, medical resource utilization; PD, pharmacodynamics; PK, pharmacokinetics; SAE, serious adverse event; SF-36 v2, 36-Item Short Form Health Survey version 2; TEAE, treatment-emergent adverse event.

Other secondary endpoints include safety assessments, infant development (as measured by Bayley Scales of Infant and Toddler Development 35 ), and immunogenicity of nipocalimab. Safety assessments for the maternal participants and neonates/infants include monitoring for treatment-emergent adverse events, including those leading to discontinuation of study intervention, as well as fetal bleeding, serious adverse events, and adverse events of special interest.

Selected exploratory endpoints include patient-reported outcome assessments (i.e., 36-Item Short Form Health Survey version 2 acute 36 and EuroQol 5-Dimension 5-Level Questionnaire 37 38 ) throughout treatment and postnatal follow-up for the maternal participants and a caregiver-reported assessment (i.e., Infant health-related Quality of life Instrument 39 40 ) during the first year of life for the infants. Other assessments include gross and microscopic placental examinations, neonatal/infant immune system development (as measured by immunoglobulin levels), and pharmacokinetics and pharmacodynamics of nipocalimab.

### Statistical Analyses

A planned sample size of approximately 50 maternal participants with at-risk FNAIT pregnancies across cohorts (4:1 randomization) was determined based on reported outcomes of weekly 1 g/kg IVIG antenatal treatment in standard-risk FNAIT pregnancies and a phase 2 study of nipocalimab in at-risk EOS-HDFN pregnancies. 30 41 42 43 This sample size is expected to provide approximately 40 to 50 participants in the standard-risk FNAIT stratum of the HPA-1a cohort, as HPA-1a is the most prevalent population at risk of FNAIT. The precision for various outcomes in the primary endpoint is based on the total sample size of the standard-risk FNAIT stratum and the observed proportion of events. Precision estimates were calculated for the standard-risk FNAIT stratum within the HPA-1a-incompatible cohort (approximately 45 of 50 participants). These estimates assume that the proportion of participants meeting the primary endpoint is 8% in the nipocalimab group 30 and 11% in the IVIG group. 41 42 43 Estimates for both groups and the resulting precision are shown in Supplementary Table S1 (available in the online version). Precision estimates for the high-risk FNAIT stratum will be analyzed separately. Efficacy analyses will be done independently for each cohort and stratum. Adverse events will be reported by participants and pooled across groups in the safety analyses. No formal hypothesis testing or interim analysis is planned for this study.

## Discussion

Although IVIG, with or without prednisone, is used for antenatal treatment of FNAIT in many countries, IVIG dose regimens vary across regions. There is also a lack of consensus regarding the use of prednisone with IVIG in high-risk pregnancies and whether to treat standard-risk HPA-5b-alloimmunized pregnancies. 7 12 19 20 21 22 While IVIG, with or without prednisone, is often effective, approximately 20 to 40% (range: 8–87%) of fetuses do not respond to antenatal IVIG and continue to have severe thrombocytopenia with platelet counts below 50 × 10 ^9^ /L. 23 41 42 43 44 There remains a substantial unmet need to improve upon IVIG due to high maternal intolerance and impaired quality of life, primarily due to headache, long treatment times, and the need for premedication. 16 17 18 45

Nipocalimab is the only therapy currently in clinical development for the treatment of alloimmunized pregnant individuals at risk for FNAIT. Nipocalimab targets the underlying pathology of FNAIT by binding to FcRn, leading to a dual effect. Specifically, nipocalimab binds to FcRn in the placenta, blocking placental IgG transfer, and also binds to FcRn in maternal endothelial cells and macrophages, blocking IgG recycling and thereby lowering circulating maternal IgG alloantibodies. 30 31

FREESIA-3 is an open-label trial and the first designed to evaluate treatment with either nipocalimab or an IVIG and prednisone reference arm in pregnant individuals at risk of FNAIT. The assessments of efficacy and safety in the FREESIA-3 study of nipocalimab align with standard practices for monitoring FNAIT outcomes, thus delivering clinically practical information. The primary composite endpoint encompasses typical clinical outcomes relevant to patients with FNAIT, including death, adjudicated severe bleeding in utero up to the first week postbirth, or low platelet count at birth (<30 × 10 ^9^ /L) in a fetus/neonate. Given that HPA-1a and/or HPA-5b subtypes are among the most prevalent, 1 5 7 enrollment in the study is limited to pregnancies complicated by FNAIT due to these HPA subtypes. By including participants with the HPA-5b subtype, the study will explore the potential effects of nipocalimab on FNAIT outcome in participants with HPA subtypes other than HPA-1a/b, in which the clinical outcomes are less well defined. In addition to evaluating efficacy and safety, this study may provide valuable insights regarding the patient experience with treatments and their implementation in clinical practice (e.g., infusion duration, steroid dose, safety).

As predicted based on its mechanism of action, nipocalimab has been associated with self-limited and recoverable reductions in maternal serum IgG concentrations (approximately 85% from baseline with the weekly 45 mg/kg IV nipocalimab dose) and low cord blood IgG. 30 Therefore, several strategies are implemented to mitigate the potential risk of infections due to decreased/low IgG in this study. First, individuals who have severe acute or chronic infections requiring antibiotics, have serum total IgG < 6 g/L, or receive or need to receive a live virus vaccine during the study or within 8 weeks after the last dose are not eligible to participate. Second, participants will be continuously monitored for any signs or symptoms of infection during the study. Third, all infants with low IgG concentrations at 52 weeks of life will be followed until the IgG levels are normalized or a stable condition is reached, with administration of IVIG permitted at the discretion of the investigator. If IgG concentrations are < 3 g/L at or after 52 weeks of life, a pediatric immunologist will be consulted. Lastly, tetanus vaccine response at 52 weeks of life will be obtained to assess immune function in both maternal participants and neonates/infants. Based on the results from completed clinical studies of nipocalimab in multiple IgG autoantibody-mediated diseases to date, nipocalimab did not increase the incidence, severity, or duration of infection. 30 31 46 47 48 49 However, further investigation is required in both maternal participants and especially neonates/infants.

A major strength of this study is the generalizability of the study results, given the randomization with a contemporaneous reference arm. Potential limitations of this study include enrollment challenges due to the rarity of FNAIT, requirements of the study design (e.g., pregnant and an estimated GA of 13 to 18 weeks, current pregnancy with the presence of maternal anti-HPA-1a and/or anti-HPA-5b alloantibody and an HPA-1a- and/or HPA-5b-positive fetus, and history of ≥ 1 prior pregnancy with FNAIT), the small number of participants in the contemporaneous study group, and potential geographic barriers (e.g., distance from the study site). Therefore, multiple study sites specialized in maternal-fetal medicine have been identified globally as study sites for the FREESIA-3 trial to enroll a sufficient number of eligible participants to facilitate interpretation of the efficacy and safety data.

## Conclusion

The FREESIA-3 trial is an ongoing, open-label, multicenter, randomized, phase 3 study designed to evaluate the efficacy and safety of nipocalimab as a noninvasive intervention for antenatal treatment in FNAIT pregnancies. The outcomes from this study will provide information on the efficacy and safety of nipocalimab in pregnant individuals and their infants with FNAIT.
