# Supplementary material for: Design of a Phase 3, Multicenter, Randomized, Open-Label Study of Nipocalimab or IVIG and Prednisone in Pregnancies at Risk for Fetal and Neonatal Alloimmune Thrombocytopenia
Source: Am J Perinatol. 2025 Dec 12;43(10):1335–44. doi: 10.1055/a-2753-9323 (PMC13345697; doi:10.1055/a-2753-9323)
Supplement: Supplementary file 1 — Supplementary Material [file 10-1055-a-2753-9323_29106559.pdf]

Supplementary Information

Supplementary Table 1. Representative estimate of precision for various outcomes for the primary endpoint calculated for the HPA-1a cohort standard-risk stratum (approximately 45 of 50 participants).

Total sample size	Treatment	Treatment sample size	Predicted number of events	Observed proportion	Clopper-Pearson 90% CI	Clopper-Pearson 95% CI
N = 45	Nipocalimab	36	3	8.3%	2.3%-20.2%	1.8%-22.5%
			2	5.1%	1.0%-16.5%	0.7%-18.7%
	IVIG with prednisone	9	1	11.1%	0.6% - 42.9%	0.3%-48.2%
			0	0%	0.0%-28.3%	0.0%-33.6%

CI, confidence interval; HPA, human platelet antigen; IVIG, intravenous immunoglobulin.
